# High-resolution transcription atlas of the mitotic cell cycle in budding yeast

**DOI:** 10.1186/gb-2010-11-3-r24

**Published:** 2010-03-01

**Authors:** Marina V Granovskaia, Lars J Jensen, Matthew E Ritchie, Joern Toedling, Ye Ning, Peer Bork, Wolfgang Huber, Lars M Steinmetz

**Affiliations:** 1 EMBL - European Molecular Biology Laboratory, Department of Genome Biology, Meyerhofstr. 1, D-69117 Heidelberg, Germany; 2 Novo Nordisk Foundation Center for Protein Research, Faculty of Health Sciences, University of Copenhagen, Blegdamsvej 3b, 2200 Copenhagen N, Denmark; 3 Department of Oncology, University of Cambridge, CRUK Cambridge Research Institute, Li Ka Shing Centre, Robinson Way, Cambridge, CB2 0RE, UK; 4 Bioinformatics Division, The Walter and Eliza Hall Institute of Medical Research, 1G Royal Parade, Parkville, Victoria 3052, Australia; 5 EMBL - European Bioinformatics Institute, Welcome Trust Genome Campus, Hinxton, Cambridge, CB10 1SD, UK; 6 Plant Biochemistry Lab, Faculty of Life Sciences, University of Copenhagen, Thorvaldsensvej 40, 1871 Frederiksberg C, Denmark

## Abstract

**Background:**

Extensive transcription of non-coding RNAs has been detected in eukaryotic genomes and is thought to constitute an additional layer in the regulation of gene expression. Despite this role, their transcription through the cell cycle has not been studied; genome-wide approaches have only focused on protein-coding genes. To explore the complex transcriptome architecture underlying the budding yeast cell cycle, we used 8 bp tiling arrays to generate a 5 minute-resolution, strand-specific expression atlas of the whole genome.

**Results:**

We discovered 523 antisense transcripts, of which 80 cycle or are located opposite periodically expressed mRNAs, 135 unannotated intergenic non-coding RNAs, of which 11 cycle, and 109 cell-cycle-regulated protein-coding genes that had not previously been shown to cycle. We detected periodic expression coupling of sense and antisense transcript pairs, including antisense transcripts opposite of key cell-cycle regulators, like *FAR1 *and *TAF2*.

**Conclusions:**

Our dataset presents the most comprehensive resource to date on gene expression during the budding yeast cell cycle. It reveals periodic expression of both protein-coding and non-coding RNA and profiles the expression of non-annotated RNAs throughout the cell cycle for the first time. This data enables hypothesis-driven mechanistic studies concerning the functions of non-coding RNAs.

## Abstract 2

A high resolution strand-specific transcriptional atlas of the budding yeast mitotic cell cycle, including both mRNA and non-coding RNA profiles.

## Background

Genome-wide transcriptome analyses in humans [1-5], mouse [6], *Drosophila melanogaster *[7,8], *Arabidopsis thaliana *[9], and fission and budding yeast [10-12] have provided evidence for widespread expression of non-coding RNAs (ncRNAs) from intergenic as well as protein-coding regions (for example, antisense or intron-derived transcripts). ncRNAs have been implicated in regulation of chromatin structure, DNA methylation, transcription, translation, as well as RNA silencing and stability [2,13-15].

Extensive transcription of intergenic regions and the antisense strands of hundreds of annotated protein-coding genes occurs in budding yeast, despite it lacking vestiges of the protein machinery required for microRNA or small interfering RNA processing [11,16-18]. It is not clear to what extent these RNAs are functional [19], but several have been shown to regulate transcription, acting through either transcriptional interference or epigenetic modifications. Examples of transcriptional interference are *SRG1*, a ncRNA transcribed in *cis *across the promoter of *SER3 *[20,21], and the antisense transcript of *IME4 *[22], whereas the antisense transcripts of *PHO5 *[23], *PHO84 *[24], transposable element *Ty1 *[25] and *GAL10-ncRNA *[26] function through epigenetic modification. For most newly discovered ncRNAs, the biological roles and mechanisms of action remain unknown. To unravel the functions of ncRNAs in yeast, it is informative to characterize them in the context of a robustly regulated and well-understood cellular process, such as the mitotic cell cycle, in which regulatory roles of ncRNAs have not been studied extensively.

The cell cycle orchestrates virtually all cellular processes - metabolism, protein synthesis, secretion, DNA replication, organelle biogenesis, cytoskeletal dynamics and chromosome segregation [27] - and diverse regulatory events depend on the maintenance of its periodicity. Between 400 and 800 periodically expressed protein-coding genes have been identified in the mitotic cell cycle and the genomic binding sites of transcription factors that control phase-specific expression of these genes have been mapped in genome-wide location analyses [28-30]. In addition to transcriptional regulation, strict timing of cell-cycle progression is ensured by post-translational regulation. This includes post-translational modifications, targeted protein degradation and indirect regulation via interactions with cell-cycle-regulated proteins [31].

To investigate the global cell cycle regulation of all transcripts, we measured high-resolution, strand-specific tiling microarray profiles of RNA expression during the *Saccharomyces cerevisiae *cell cycle. In contrast to previous studies [29,30], which only interrogated annotated features within the genome without resolving strand specificity, the fine spatial and temporal resolution of our dataset enabled us to look at the whole transcriptome on both strands, including non-coding RNAs (both away from coding genes and in antisense position), complex transcription architecture of protein-coding genes, alternative transcription start and polyadenylation sites, splicing, and differential regulation of sense and antisense transcripts. Our data reveal cell-cycle-regulated non-coding genes, complex expression coupling between sense and antisense transcripts, as well as over 100 protein-coding genes that were not previously known to cycle.

## Results and discussion

### Detecting periodic transcripts

We monitored genome-wide cell-cycle-regulated expression at 5-minute intervals for up to three cell division cycles, using whole-genome tiling arrays [11]. The array is unique in interrogating every base pair in the genome on average six times and providing an 8-bp resolution for strand-specific probes. Two independent synchronization methods were used in order to obtain synchronous cultures (see Materials and methods; Additional file 1). Late G1 phase arrest was induced by exposure of *bar1 *cells to alpha factor, and by raising the temperature to 38°C for temperature-sensitive *cdc28-13 *mutant cells. Expression profiles for all genomic regions are provided in a database that is searchable by gene symbol or chromosomal coordinate [32].

To identify all transcribed sequences, we segmented along-chromosome expression profiles, applying an adaptation of the method described by Huber *et al*. [33] (see Materials and methods). In addition to protein-coding transcripts and infrastructural RNAs, we registered abundant expression of unannotated non-coding RNAs (Additional file 2). These unannotated expressed features comprise 523 antisense transcripts opposite protein-coding regions and 135 intergenic transcripts (Additional file 3). The length distribution of ORFs in these unannotated transcripts is within the range that is expected by chance. Hence, we find no evidence for the unannotated transcripts to be protein coding.

The average segment levels from each time-point were analyzed for periodic expression by two different computational methods [34,35], as well as by visual inspection. The aim of this combination of methods was accurate and sensitive detection of cell-cycle-regulated transcripts (see Materials and methods). In order to validate our approach, we compared our gene list of periodic protein-coding genes to a benchmark set that comprised all known cell-cycle-regulated genes identified in single-gene experiments [35,36]. Our individual cdc28 and alpha-factor datasets were each better than most of the available ones [28-30] (Additional file 4). Furthermore, our combined list of periodic protein-coding genes, despite being based on just two experimental datasets, performed almost as well in identifying the benchmark set of genes as that of Gauthier *et al*. [37], which integrated all available genomic datasets of cell-cycle-regulated genes performed to date (Additional file 4). Thus, our dataset and analysis method reproduced the previous data on cycling protein-coding genes.

**Figure 1:**
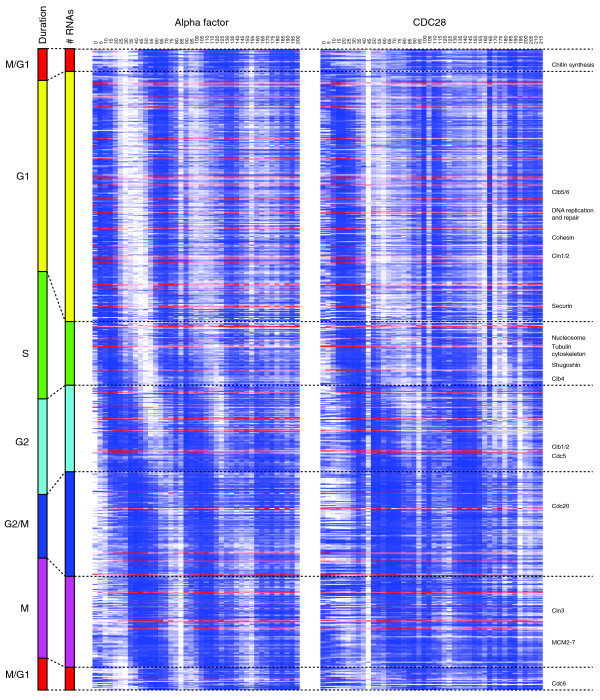
**Gene expression profiles ordered by expression peak times**. CDC28 and alpha-factor panels show the expression profiles for all identified cell cycle-regulated genes, including 598 protein-coding genes, 37 unannotated antisense transcripts and 11 intergenic transcripts, ordered by their peak times. Profiles for annotated ORFs are graded in blue; all non-coding RNAs are graded in red. Each column of the two time-course panels represents a single experimental 5-minute time-point. The scales on the left display the relative duration and number of transcripts expressed in each phase. Key cell-cycle-regulated genes are indicated on the right side. In each row, white and dark blue (or dark red for the non-coding RNAs) represent the minimum and maximum expression levels, respectively, of the corresponding transcript. Intermediate values are shown by colors that scale linearly over the range.

**Figure 2:**
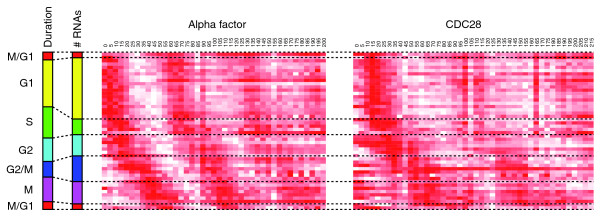
**Gene expression profiles for all identified cell-cycle-regulated ncRNAs ordered by their expression peak times**. Each column of the CDC28 and alpha-factor time-course panels represents a single experimental 5-minute time-point. The scales on the left display the relative duration and number of transcripts expressed in each phase. In each row, white and dark red represent the minimum and maximum expression levels, respectively, of the corresponding transcript. Intermediate values are shown by colors that scale linearly over the range.

Altogether, 598 periodic mRNAs, 37 cycling antisense RNAs, and 11 cycling intergenic transcripts were identified and ranked according to their peak time of expression (Figure 1; Additional file 5). Non-coding periodic transcripts were expressed in all cell-cycle phases (Figure 2; see Additional file 6 for the determination of the boundaries of the cell cycle phases). Overall, the peak times of antisense periodic expression were consistent with the waves of expression of periodic protein-coding genes [38]. To characterize the newly discovered periodic ncRNAs, we overlapped them with regions of conserved RNA secondary structure [39]. Despite their cell-cycle-regulated expression, the unannotated intergenic and antisense ncRNAs had little secondary structure (Additional file 6). Conversely, infrastructural ncRNAs, comprising tRNAs, rRNAs, small nuclear and small nucleolar RNAs, were highly structured but were not periodically expressed.

### Cell-cycle-regulated expression of unannotated non-coding RNAs

Studies in mammalian cells have suggested that antisense RNAs could regulate gene expression of their sense counterparts, whereby sense and antisense transcripts often exhibit expression correlation patterns [40,41] and overlap in opposite directionality [42]. We thus analyzed antisense RNAs in the context of the sense-antisense pairs (SAPs) of which they are a part. We categorized the pairs into four classes based on their expression coupling: 13 periodic antisense transcripts opposite periodic sense transcripts; 24 periodic antisense transcripts opposite non-periodic sense transcripts; 43 non-periodic antisense transcripts opposite periodic sense transcripts; and 443 non-periodic antisense and sense transcript pairs (Additional file 7).

The 13 periodic antisense transcripts opposite periodic sense transcripts were further subdivided based on the relative timing of expression of the sense and antisense transcripts. Considering the absolute difference between their expression peak times, two pairs (*ALK1 *and *HSL1*) cycle in-phase, whereas seven (*CTF4*, *FAR1*, *HMS2*, *TAF2*, *TIP1*, *YNL300W *and *YPL162C*) show anti-correlated expression (Additional file 8). Expression profiles of the other four SAPs (*PRY3*, *YLR050C*, *YMR253C *and *YPL230W*) had phase shifts between 0 and π.

**Figure 3:**
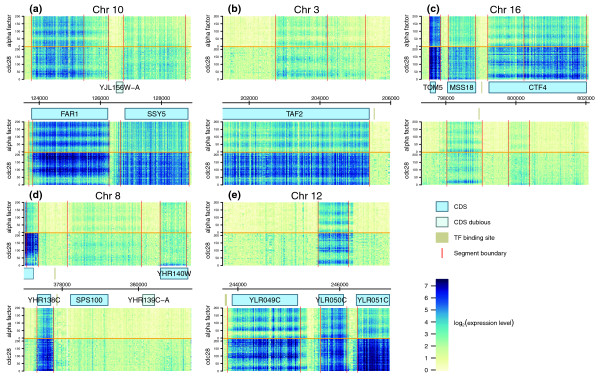
**Expression for sense and antisense transcripts**. Heatmaps of expression for sense and antisense transcripts of **(a) ***FAR1*, **(b) ***TAF2*, **(c) ***CTF4*, **(d) ***SPS100 *and **(e) ***YLR050C*. Each horizontal line represents a single experimental time-point. The unit of the time axis (vertical) is minutes. The horizontal axis in the center of each panel represents genomic coordinates, and annotated coding genes are indicated by blue boxes. The heatmap in the upper half of each panel represents signal on the Watson strand, the one in the lower half signal on the Crick strand. The horizontal orange lines separate alpha-factor (above the line) and Cdc28 (below the line) experimental datasets. Vertical red lines show the segment boundaries.

Remarkably, several genes encoding important cell cycle regulators fall within the categories listed above (Figure 3a-c). Among them, *FAR1 *is important for mating pheromone-induced growth arrest and, together with cyclins *CLN2 *and *CLN3*, plays one of the key roles in the G1/S transition [43]. *FAR1 *is expressed at the M/G1 transition and needs to be shut down in late G1 for the cell to pass the G1/S checkpoint. Its antisense RNA peaks starting from the late G1 phase and throughout the G1/S transition, when Far1 protein should not be present. *TAF2*, which is involved in transcription initiation, is expressed in late M and early G1 phase; its antisense transcript peaks in late G1 and further into S phase. The sense and antisense transcripts of *CTF4*, which shapes and maintains chromatin structure to ensure the passage through the S-phase checkpoint [44], are expressed in an anti-correlated manner, peaking in the G1/S and G2/M transitions, respectively. The *CTF4 *sense transcript appears to be transcribed from a bidirectional promoter shared with the antisense transcript of the neighboring gene, *MSS18 *(Additional files 6 and 9). Together these expression patterns suggest that some of the antisense transcripts may play a role in cell-cycle regulation.

We analyzed Gene Ontology (GO) categories of genes overlapped by antisense transcripts. Most of the protein-coding messages opposite the 37 periodic antisense transcripts (13 + 24) fall into GO categories linked with the process of cell division, including cell wall and organelle organization and biosynthesis, regulation of transcription, signal transduction and protein modification, carbohydrate metabolic processes, and cell cycle (Additional file 10). Surprisingly, 15 of the 37 sense transcripts are of unknown function. We carried out a similar analysis for the 43 non-periodic antisense transcripts opposite periodic sense transcripts. As expected, most of these cycling sense messages fall into cell-cycle-related GO categories, including genes involved in bud site selection and polarization (*BUD9*, *GIC1*), daughter cell separation from the mother (*DSE2*, *CTS1*), cell wall proteins, and so on (Additional file 7). Analysis of GO categories for the remaining 443 non-periodic SAPs did not show enrichment in any particular category, although almost a quarter of the genes have unknown function (Additional file 11).

We observed a statistically significant correlation (*P *< 0.002; 5 × 4 contingency table; χ^2 ^test) between the overlap patterns of the sense and antisense transcripts and the relationship of their expression profiles (Additional file 12). Altogether we distinguished five types of overlap within a given SAP: antisense transcript contains the transcribed message of its sense counterpart; the antisense transcript is contained within the sense transcript; the antisense transcript overlaps either the 3' or the 5' end of its sense partner; and the antisense transcript overlaps two distinct sense transcripts. The following patterns of overlap were over-represented compared to what was expected by chance. In 8 out of 13 periodic antisense transcripts opposite periodic sense transcripts, the antisense transcript is mainly contained within the protein-coding message; 2 of these 8 cycle in-phase, and 6 display opposite-phase expression. For 5 of 24 SAPs in which only the antisense transcript cycles, the antisense transcript contains the complete sense message, and for another 5, it overlaps 2 sense transcripts. In 15 of the 43 pairs in which only the sense message is cell cycle regulated, the antisense transcript overlaps the 5' end of the mRNA and in many cases extends further upstream.

To investigate sense and antisense expression in more detail, we also searched for putative TF binding sites (Additional file 6) and supported these predictions with the existing ChIP-chip data. TF binding site analyses are inherently non-strand-specific; however, our data on the temporal expression of the sense and antisense transcripts yield clues to the regulation of strand-specific expression. For example, ChIP-chip data and our motif analysis for *FAR1 *suggest binding of both the M-phase TF Mcm1 [45] and the G1/S TF SBF [46] within the region spanned by 600 bases before and after the transcript. This evidence for SBF regulation of *FAR1 *contradicts the timing of expression of the sense transcript since *FAR1 *is expressed at the M/G1 transition and needs to be shut down in late G1. Our data show late-G1-specific expression of the *FAR1 *antisense transcript, thus providing a putative explanation for the presence of the TF binding site for SBF. Overall, our analyses indicate that the cycling unannotated transcripts have binding sites for the same set of TFs that drive sense transcription during the cell cycle (Additional file 6).

Altogether, 135 unannotated intergenic transcripts were detected in our dataset. Of these, 11 oscillate with mitotic progression (Additional files 5; Additional file 13c). As for the antisense transcripts, their peak in expression follows the waves of excitation in mitotic progression observed for protein-coding genes [38]. To elucidate the role of these intergenic transcripts in cell cycle regulation, deletion strains for 10 of the 11 unannotated periodic transcripts were generated in both strain backgrounds. Growth curves of the deletion strains did not show significant lagging in cell doubling time after asynchronous growth in rich media for 28 hours at 30°C and 37°C. Lack of phenotype is consistent with our previous observations for the unannotated intergenic transcripts detected from asynchronous culture [11]. This suggests that their deletion phenotypes have more subtle effects than those of many protein-coding genes.

### Cell cycle-regulated protein-coding genes

**Figure 4:**
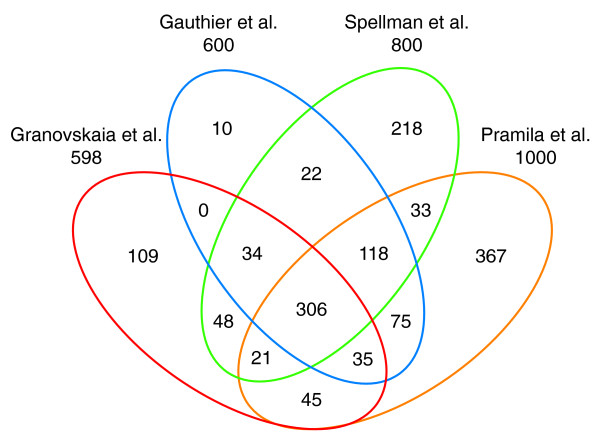
**Venn diagram displays the overlap of our list of identified cell cycle-regulated protein-coding genes with the lists determined by the previous studies of Gauthier *et al***. [37], **Pramila *et al*. **[29], **and Spellman *et al*. **[30]. The overlap shows that we find an additional 223 genes not identified by Gauthier *et al*., among which 109 are unique to our dataset and were not previously defined by the other studies.

Previous studies have identified a large number of annotated periodic transcripts. Compared to the integrated dataset of Gauthier *et al*. [37], our list contains 223 additional periodic protein-coding genes, of which 109 were also not identified by Pramila *et al*. [29] and Spellman *et al*. [30] (Figure 4; Additional file 14). Only 3 of the 109 have been shown to be periodically expressed in small scale experiments [47]. GOslim analysis [48] showed that the biological function is unknown for 35 of these 109 genes, whereas 41 perform functions directly or indirectly associated with the regulation of the cell cycle, such as organelle organization and biogenesis, cytoskeleton organization and biogenesis, ribosome biogenesis and assembly, and so on (Additional file 15).

Of the 598 periodically expressed protein-coding genes, just 7 contain an intron according to the *Saccharomyces *Genome Database annotation: *CIN2*, *MOB1*, *PMI40*, *RFA2*, *SRC1*, *TUB1*, and *USV1*. This is due to the fact that many of the budding yeast introns reside within genes that encode ribosomal proteins [48]. In addition, none of the introns in periodically expressed genes show signs of phase-specific splicing; hence, in contrast to meiosis in budding and fission yeast [49,50], we see no evidence for a regulatory role of splicing in the mitotic cell cycle of budding yeast.

## Conclusions

Our data provide 5-minute resolution strand-specific profiles of temporal expression during the mitotic cell cycle of *S. cerevisiae*, monitored for more than three complete cell divisions. The resulting atlas for the first time comprehensively maps the expression of non-annotated regions transcribed in mitotic circuitry, measures the expression coupling of protein-coding and non-coding transcript pairs and reveals strand specificity of transcription regulation. Furthermore, it unravels complex architectures of the mitotic transcriptome, such as splicing and alternative transcription start and polyadenylation sites, and extends the set of previously reported cell-cycle-regulated genes by 109 protein-coding genes.

The abundance of antisense expression across the genome raises the question of whether it represents opportunistic 'ripples of transcription' through active chromatin regions, or whether it is a regulated overlap between the transcripts [51]. An evolutionary analysis of genes with overlapping antisense partners across a number of eukaryotic genomes has indicated that the sense-antisense arrangement is more highly conserved than expected if it were random 'leakage' of the transcription machinery [52].

Regulatory roles for a few antisense transcripts have been documented in yeast [20-25], yet it is still debated what proportion of ncRNAs are functional [19]. Our dataset reveals that most cycling antisense transcripts are located opposite genes with cell-cycle-related functions. Antisense transcripts may regulate the corresponding functional sense transcripts through several molecular mechanisms, which can be speculated from the mutual expression pattern of the two transcripts [53]. For example, transcriptional interference or antisense-dependent inhibitory chromatin remodeling may give rise to the anti-correlated expression of sense and antisense transcripts, as is observed for more than half of the 13 periodic SAPs. For the 24 cases where the antisense transcript cycles while the sense transcript is stably expressed, the periodic antisense transcript may putatively mask the sense transcript, thereby conferring periodic regulation at the level of translation. Through the same mechanism, the 43 stably expressed antisense transcripts may dampen stochastic fluctuation of sense messages by setting a threshold above which the sense expression must rise [53]. Alternatively, stably expressed antisense transcripts could mediate activatory chromatin remodeling that maintains the chromosomal region in a transcriptionally activatable/repressible state and thereby facilitate expression regulation of the periodic sense transcript. Indeed, more than one-third of the 43 stably expressed antisense opposite cell-cycle-regulated mRNAs overlap with the 5' UTRs. Altogether, the sense-antisense expression coupling may help to narrow down molecular mechanisms through which a specific antisense transcript exerts its function. Our high-resolution, unbiased expression atlas of the budding yeast cell cycle is thus a resource with which to unravel a potential additional level of the cell cycle regulatory circuit, as well as to study the periodic expression of protein-coding transcripts at a fine temporal and spatial resolution. The dataset provides a link between genomic approaches and hypothesis-driven mechanistic research with regard to the functions of ncRNAs.

## Materials and methods

### Yeast strains and cell cycle synchronization

W101 (50 ml; MAT**a ***ade2-1 trp1-1 leu2-3*, *112 his3-11*, *15 ura3 can1-100 *[psi1]) background temperature-sensitive *cdc28-13 *mutant *S. cerevisiae *strain K3445 (YNN553) was grown for approximately 8 to 10 hours in rich yeast-extract/peptone/dextrose (YPD) in a shaking water bath at 25°C and diluted in 3 × 1.6 liter cultures for overnight growth in an air incubator at 25°C. The following morning the cultures of OD600 approximately 0.2 were mixed together, distributed into 45 × 100 ml samples and arrested in late G1 at START by shifting the temperature from 25°C to 38°C. After 3.5 hours, the cells were transferred back to permissive temperature to re-initiate cell division and samples were collected every 5 minutes for 215 minutes (equal to more than two complete cell cycles). The cultures were centrifuged and snap-frozen in liquid nitrogen. The degree of synchrony was monitored by assessing the number of budding cells and measuring the bud size (Additional file 1). Nuclear position was determined by Hoechst staining with fluorescence microscopy (Additional file 16).

To arrest *bar1 *strain DBY8724 (MAT**a ***GAL2 ura3 bar1::URA3*) [30] in G1 at START, alpha-factor pheromone was added to a final concentration of 600 ng/ml. After 2 hours of arrest, cells were released by washing and recovered in fresh preconditioned medium to facilitate initiation of mitosis. Samples were collected every 5 minutes for 200 minutes (equal to three cell cycles). The degree of synchrony was monitored by assessing the number of budding cells. Nuclear position was determined by Hoechst staining with fluorescence microscopy.

### Total RNA extraction, poly(A)-RNA enrichment, cDNA synthesis and labeling

Total RNA was isolated from the culture corresponding to each time-point by the standard hot phenol method [11]. Poly(A)-RNA was enriched from 1 mg of total RNA by a single passage through the Oligotex Oligo-dT Column (Qiagen, Hilden, Germany). Poly(A)-RNA was treated with RNase-free DNaseI (Ambion's Turbo DNA-free Kit, Foster City, CA, USA) for 25 minutes at 37°C according to the manufacturer's instructions and subsequently reverse transcribed to single-stranded cDNA for microarray hybridization. Each 200 μl reverse transcription reaction was carried out in duplicate and comprised 6 μg of poly(A)-RNA, 3 μg random hexamers (RH6), 1 μl of 6 mg/ml Actinomycin D (ActD), 0.4 mM dNTPs containing dUTP (dTTP:dUTP = 4:1), 40 μl 5× first strand synthesis buffer (Invitrogen, Karlsruhe, Germany), 20 μl 0.1 M dithiothreitol (Invitrogen), and 1,600 units of SuperScript II (Invitrogen). The synthesis was carried out at 42°C for 1 h and 10 minutes, followed by reverse transcriptase inactivation at 70°C for 10 minutes. Poly(A)-RNA and RNA in heteroduplex with cDNA were digested by a mixture of 3 μl of RNAseA/T cocktail (Ambion) and 3 μl of RNAseH (Invitrogen) for 15 minutes at 37°C followed by inactivation of the enzymes for 15 minutes at 70°C. Replicate cDNA samples were further applied to the Affy Clean-up column (Affymetrix, Santa Clara, CA, USA), eluted together in 30 μl DEPC-H_2_O and quantified. Purified cDNA (3.3 μg of each 5-minute time-point sample) was fragmented and labeled with WT Terminal Labeling Kit (Affymetrix) according to the manufacturer's instructions and then hybridized to tiling arrays.

### Genomic DNA preparation

For DNA hybridization, both strains were grown in YPD media overnight to saturation in three biological replicates and whole-genomic DNA was purified using the Genomic DNA Kit (Qiagen). Genomic DNA (10 μg) was digested to 25 to 100 base fragments with 0.2 U of DNaseI (Invitrogen) in 1× One-Phor-All buffer (Pharmacia, Munich, Germany) containing 1.5 mM CoCl_2_(Roche, Mannheim, Germany) for 3.5 minutes at 37°C. After DNaseI inactivation by boiling for 10 minutes, the sample was 3' end-labeled in the same buffer by the addition of 1.5 μl of Terminal Transferase (25 units/μl; Roche) and 1.5 μl 10 mM biotin-N6-ddATP (Molecular Probes, Karlsruhe, Germany) for 2 hours at 37°C, and hybridized to the tiling array.

### Array design

The array was designed in collaboration with Affymetrix (PN 520055), as described in David *et al*. [11]. Probe sequences were aligned to the genome sequence of *S. cerevisiae *strain S288c (*Saccharomyces *Genome Database of 7 August 2005). Perfect match probes were further analyzed.

### Probe normalization and segmentation

The log-base 2 perfect match (PM) probe intensities from each array were background corrected and calibrated using the DNA reference normalization method described in Huber *et al*. [33], which was applied separately to both datasets, cdc28 and alpha-factor.

To determine the transcript boundaries in the combined dataset, a piece-wise constant model was fitted to the normalized intensities of the unique probes ordered by genomic coordinates. The basic model described in Huber *et. al*. [33] was modified to allow time-point-dependent levels. The normalized intensities (*z*_*jk*_) were modeled as:

where *μ*_*sk *_is the array-specific level of the *s*-th segment, *ε*_*jk *_are the residuals, *j *= 1, 2,., *n *indexes the probes in ascending order along the chromosome, *k *indexes the time-point (array), *t*_2_,., *t*_*S *_parameterize the segment boundaries (*t*_1 _= 1 and *t*_*S*+1 _= *n + *1) and *S *is the total number of segments. Model 1 was applied separately to each strand of each chromosome. For each chromosome, *S *was chosen such that the average segment length was 1,250 nucleotides. Change-points were estimated using a dynamic programming algorithm implemented in the tilingArray package [33].

After segmentation, the average of the probe signals within the segment boundaries was calculated for each time point. A table of segment levels is available from the supplementary materials webpage [32].

To estimate a threshold for expression, the average level over both datasets was calculated for each segment. Segments not overlapping annotated, transcribed features were used to estimate the background level as follows. A normal distribution was fit in order to determine a threshold at which the estimated false discovery rate was 0.1% [11]. For the mean of the normal distribution, we used the midpoint of the shorth (the shortest interval that covers half of the values), for the variance, the empirical variance of the lowest 99.9% of the data. Segments whose level fell below this threshold were considered not expressed.

Segments were then assigned to different categories depending on how they overlapped with annotated features as described in David *et al*. [11], with the difference of re-naming the unannotated isolated features to the unannotated intergenic. Expression values for each annotated feature were calculated as weighted averages of the overlapping segments on the same strand.

### Detection of periodic genes

We used a combination of three approaches to identify periodically expressed segments and annotated features based on the cdc28 and alpha-factor datasets: the method of Ahdesmaki *et al*. [34], which calculates *P*-values for a robust nonparametric version of Fisher's g-test [54,55], the permutation-based method of de Lichtenberg *et al*. [35], which scores genes based on both the magnitude of regulation and the periodicity of profile, and by systematic visual inspection. For the two computational methods, score cutoffs were determined based on comparison with existing benchmark sets of 113 known cycling genes identified in single-gene studies [47]. A combined list of cycling transcripts was compiled that contains all transcripts identified as cycling by at least two of the three methods. The peak time of expression for each transcript was calculated as percentage of the cell cycle duration as previously described [35]. To determine the length of the cell cycle in each experiment, the period length was optimized to fit the expression profiles for selected genes from the benchmark set.

### Analysis of protein-coding potential

To test if the ncRNAs are likely to be novel protein-coding genes, we extracted all ORFs within unannotated antisense and intergenic transcripts and compared their length distributions to what would be expected by chance. The length of an ORF was defined as the distance between a stop codon and the most upstream ATG codon. Two separate background distributions were used for antisense and intergenic transcripts, to take into account that these two types of ncRNAs have different sequence properties (k-mer frequencies), because the former are located opposite of protein-coding genes whereas the latter are located within intergenic regions. For antisense transcripts, a set of sequences with the same length distribution was sampled from the genomic regions opposite other protein-coding genes. Opposite genomic regions with matched length distribution and sequence properties were used as a background for the unannotated intergenic RNAs. The ORF length distributions observed for the antisense and intergenic transcripts were not statistically significantly different from their respective background distributions according to the Kolmogorov-Smirnov test.

### Transcription factor binding sites analysis

We used the TAMO suite [56] to identify the TFs that preferentially bind to regulatory regions of periodic non-coding transcripts. We systematically searched for binding motifs that were significantly overrepresented for the region, spanning from -600 bp upstream up to +600 bp downstream of 37 periodic unannotated antisense and 11 intergenic transcripts of interest, relative to a background set composed of all transcripts detected in the alpha-factor experiment. A benchmark set comprised 113 genes whose transcription was reported as cell cycle regulated in single-gene studies previously [47], whereas the lowest scoring 252 non-periodic antisense transcripts from the alpha-factor induced arrest dataset served as a negative control. We also performed *de novo *motif discovery on these sequences, using the combination of methods contained in the TAMO software suite. This analysis revealed no significantly overrepresented sequence motifs. We then searched for the putative TF binding sites that matched the position-specific score matrices from MacIsaac [57,58].

### Analysis of RNA secondary structure conservation

We investigated the overlap between transcripts and genomic regions with conserved secondary structure [39]. We used Steigele *et al*.'s [39] regions for cutoff 0.5. The regions were remapped to the current genome assembly using Exonerate (requiring 100% identity). The regions are strand-specific and overlap with these regions was also considered in a strand-specific way.

### Deletion strains of the periodic unannotated intergenic transcripts

We generated deletion strains with the help of PCR-based technology as described on the Stanford Yeast Deletion webpage [59] using a set of up- and downstream primers flanking the defined periodic unannotated sequence listed in Additional file 5. The growth of deletion strains was monitored in liquid media using GENios automatic microplate readers (TECAN).

## Abbreviations

ChIP: chromatin immunoprecipitation; GO: Gene Ontology; ncRNA: non-coding RNA; ORF: open reading frame; SAP: sense-antisense pair; TF: transcription factor; UTR: untranslated region.

## Authors' contributions

MVG and LMS designed research; MVG performed research; YN contributed to research; MVG, MER, LJJ, JT, WH and LMS analyzed data; MVG, LJJ, MER, WH and LMS wrote the paper; WH, PB and LMS supervised research. The authors declare that they have no conflict of interest.

## Supplementary Material

Additional file 1 A table providing control data on the synchronous division of the yeast cells. Excel sheet 1 contains a table of the number and percentage of budded cells and dividing nuclei over time with the progression of the cell cycle; sheet 2 contains a chart of these data. Click here for file

Additional file 2 A figure showing categories of expressed segments. The pie chart shows the categories and the numbers of all identified transcribed segments. The unassigned categories encompass the segments that did not meet filter criteria and were excluded from further analyses [11]; correspondingly, the filtered categories are those that did pass the filtering criteria. Click here for file

Additional file 3 A table listing antisense and novel intergenic transcripts identified in our study. Excel sheet 1 is a table of all 523 antisense transcripts, characterized by their genomic position, length and overlapping sense feature; sheet 2 is a table of all 135 unannotated intergenic transcripts, categorized by genomic position and length. Cycling intergenic transcripts are highlighted in sheet 2. Click here for file

Additional file 4 A figure showing a comparison of our dataset with the published datasets on the cell cycle in yeast. Three ROC-like plots compare: **(a) **our combined dataset with that of Gauthier *et al*. [37]; **(b) **our cdc28 dataset with the other Cdc28 datasets of Spellman *et al*. [30] and Cho *et al*. [28]; **(c) **our alpha-factor dataset with the existing alpha-factor datasets of Spellman *et al*. [30] and Pramila *et al*. [29]. The fraction of the B1 benchmark set genes identified by the various datasets is plotted as a function of gene rank. (a) Comparison of the method of de Lichtenberg *et al*. applied to our data (red line) with the comprehensive integrated dataset of Gauthier *et al*. (black line) [35]. The cross indicates our combined list, obtained by the combination of two computational methods of analyses, and curated manually. (b) Comparison of Cdc28 datasets. (c) Comparison of alpha factor-induced growth arrest datasets. The color code displays: light brown, Cho *et al*.; green, Spellman *et al*.; cyan and blue, Pramila *et al*.; black, Gauthier *et al*.; red, this study. The dotted line indicates random selection of genes. Click here for file

Additional file 5 A table listing periodic protein-coding genes, antisense and unannotated intergenic transcripts. Excel sheet 1 lists 598 periodic ORFs identified in our dataset, sheet 2 lists 37 cycling antisense transcripts, and sheet 3 lists 11 periodic unannotated intergenic transcripts. Click here for file

Additional file 6 A Word document providing supplemental data. The file provides additional information on the following sections: 1, Determination of the boundaries of the cell cycle phases; 2, Conservation analysis of non-coding RNAs; 3, Analysis of upstream regulatory elements for periodic unannotated transcripts; 4, UTR lengths; 5, Divergently transcribed periodic transcripts. Click here for file

Additional file 7 A table listing the categories of 37 periodic and 43 non-periodic antisense transcripts. Excel table sheet 1 lists 37 periodic antisense transcripts and sheet 2 lists 43 non-periodic antisense transcripts, each characterized by genomic position, length, overlapping sense feature, function of the opposite sense counterpart according to the *Saccharomyces *Genome Database, and peak time of expression (cycling 37 antisense transcripts only). Click here for file

Additional file 8 A figure showing a comparison of the relative timing of expression within 13 periodic SAPs. We calculated the peak-time difference for the periodic sense and antisense transcripts within each of the 13 cycling SAPs for the alpha-factor and Cdc28 experiments separately. A difference of 0 corresponds to in-phase expression, whereas a difference of 50 corresponds to opposite-phase expression (180 degree phase shift). We observe a good correlation between the two experiments. The shape of the symbol shows how the sense-antisense counterparts overlap. Click here for file

Additional file 9 A table listing pairs of pairs of divergent transcripts from a bidirectional promoter. Each transcript in a pair is characterized by the genomic location, category and gene name. Click here for file

Additional file 10 A figure showing GO categories of the ORFs opposite cell-cycle-regulated antisense transcripts. The x-axis displays the number of genes and the y-axis shows the names of GO categories. Click here for file

Additional file 11 A figure showing GO categories of 443 non-periodic ORFs opposite non-periodic antisense transcripts. The x-axis displays the number of genes and the y-axis shows the names of GO categories. Click here for file

Additional file 12 A contingency table for sense-antisense transcript overlap. Click here for file

Additional file 13 A figure showing heatmaps of bi-directional expression of neighboring cell cycle-regulated genes that share transcription regulatory elements. **(a) **Two neighboring ORFs: *TEL2 *and *ESP1*. **(b) **ORF and an antisense transcript of the upstream protein-coding gene: *SPT21 *and antisense counterpart of *YMR178W*. **(c) **ORF and cycling unannotated intergenic transcript: *MCD1*and upstream cycling novel transcript. The heatmap plot is explained in the caption of Figure 3. Click here for file

Additional file 14 A table listing the 109 periodic ORFs identified in our study. Click here for file

Additional file 15 A figure showing GO categories of 109 periodic ORFs unique to our dataset. The x-axis displays the number of genes and the y-axis shows the names of GO categories. Click here for file

Additional file 16 A figure showing Hoechst nuclear staining of dividing *cdc28-ts *mutant cells. Control data displaying synchronous division of the yeast cells along with the cell cycle progression. Each image represents a gallery of approximately 10 to 20 representative cells that were chosen, for the respective time-point, from different fields of view. Criteria of choice were sharpness of the image and visibility of the bud; besides these, we aimed for random selection. Click here for file
