# Supplementary figures and images for: High-resolution transcription atlas of the mitotic cell cycle in budding yeast
Source: Genome Biol. 2010 Mar 1;11(3):R24. doi: 10.1186/gb-2010-11-3-r24 (PMC2864564; doi:10.1186/gb-2010-11-3-r24)

# Categories of expressed segments

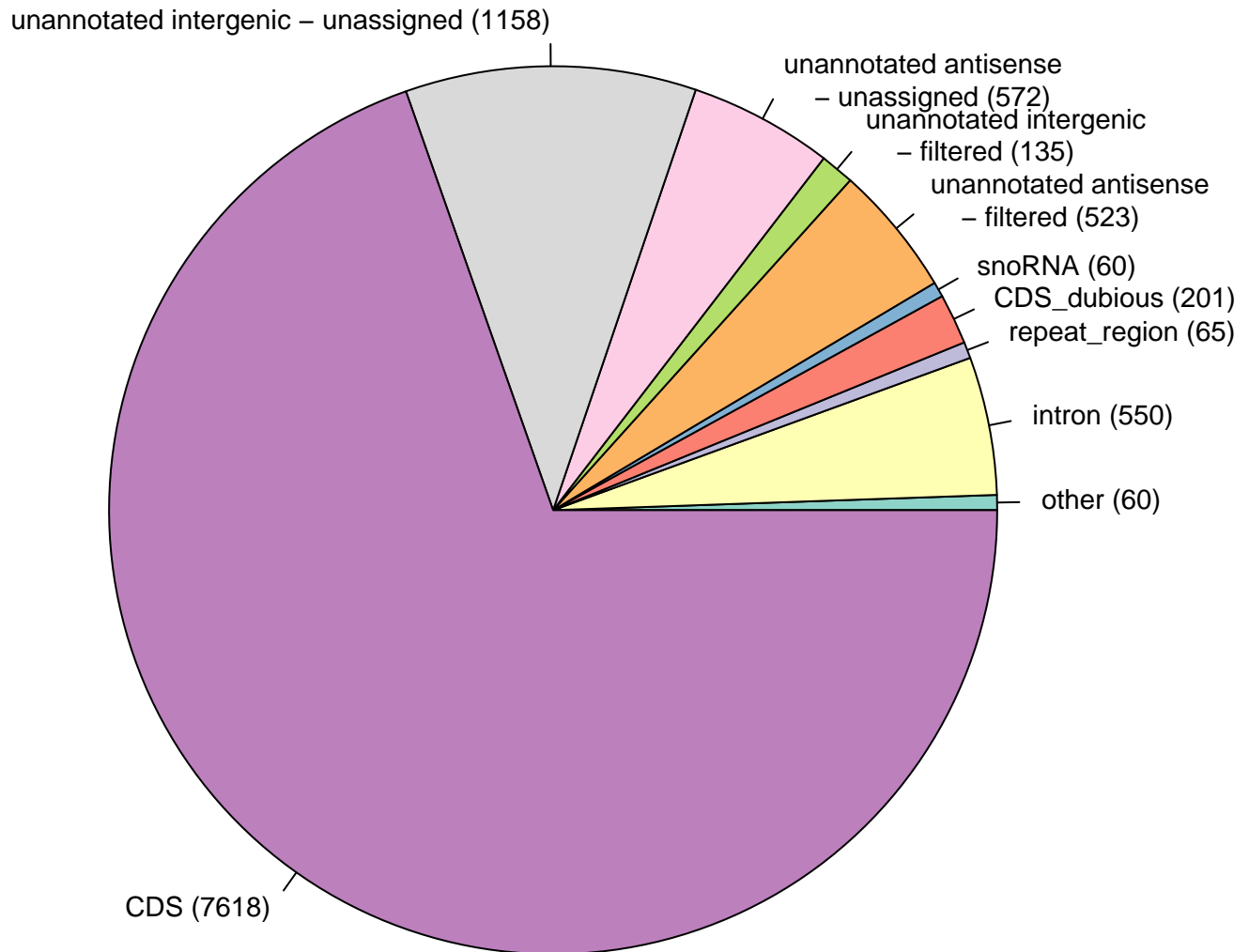

Supplement: Additional file 2 — A figure showing categories of expressed segments. The pie chart shows the categories and the numbers of all identified transcribed segments. The unassigned categories encompass the segments that did not meet filter criteria and were excluded from further analyses [11]; correspondingly, the filtered categories are those that did pass the filtering criteria. [file gb-2010-11-3-r24-S2.pdf]

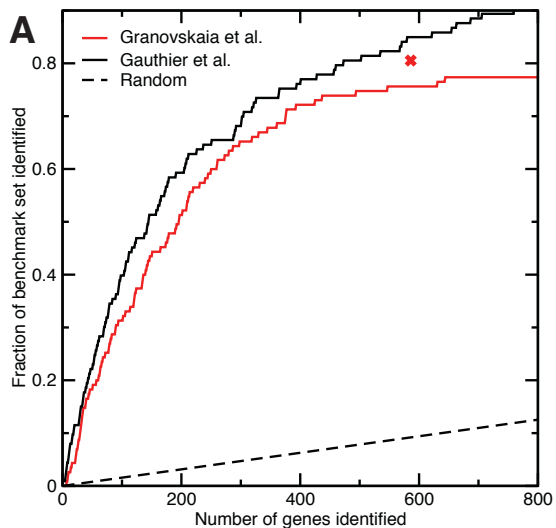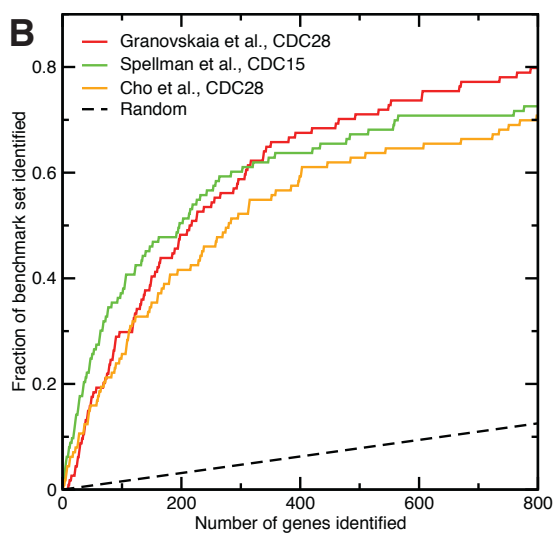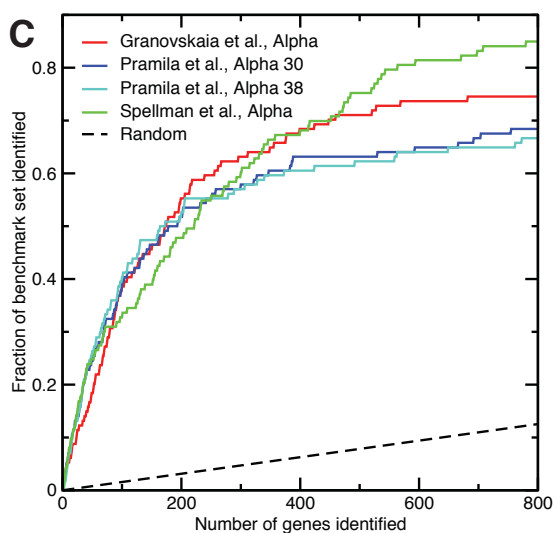

Supplement: Additional file 4 — A figure showing a comparison of our dataset with the published datasets on the cell cycle in yeast. Three ROC-like plots compare: (a) our combined dataset with that of Gauthier et al. [37]; (b) our cdc28 dataset with the other Cdc28 datasets of Spellman et al. [30] and Cho et al. [28]; (c) our alpha-factor dataset with the existing alpha-factor datasets of Spellman et al. [30] and Pramila et al. [29]. The fraction of the B1 benchmark set genes identified by the various datasets is plotted as a function of gene rank. (a) Comparison of the method of de Lichtenberg et al. applied to our data (red line) with the comprehensive integrated dataset of Gauthier et al. (black line) [35]. The cross indicates our combined list, obtained by the combination of two computational methods of analyses, and curated manually. (b) Comparison of Cdc28 datasets. (c) Comparison of alpha factor-induced growth arrest datasets. The color code displays: light brown, Cho et al.; green, Spellman et al.; cyan and blue, Pramila et al.; black, Gauthier et al.; red, this study. The dotted line indicates random selection of genes. [file gb-2010-11-3-r24-S4.pdf]

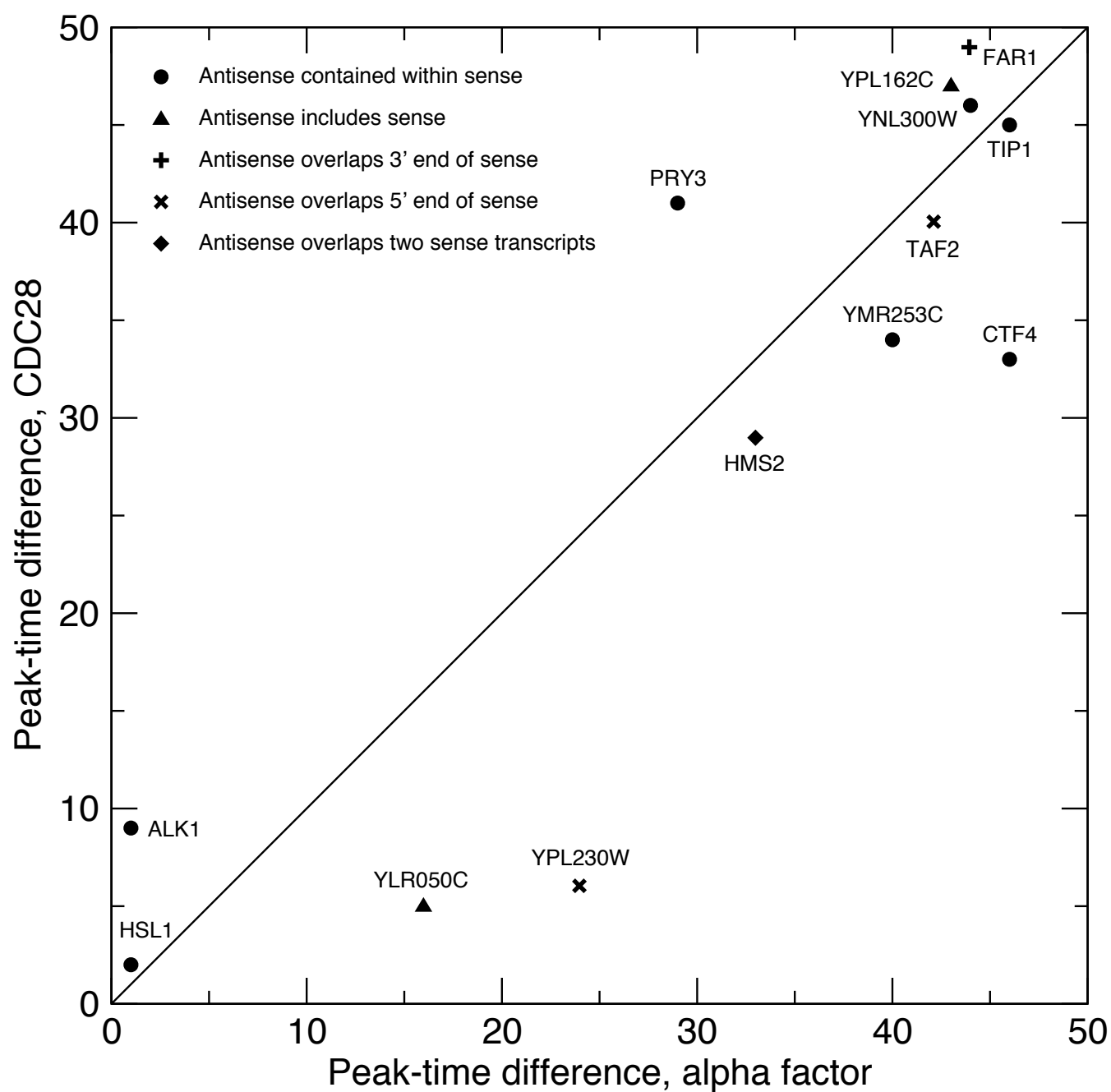

Supplement: Additional file 8 — A figure showing a comparison of the relative timing of expression within 13 periodic SAPs. We calculated the peak-time difference for the periodic sense and antisense transcripts within each of the 13 cycling SAPs for the alpha-factor and Cdc28 experiments separately. A difference of 0 corresponds to in-phase expression, whereas a difference of 50 corresponds to opposite-phase expression (180 degree phase shift). We observe a good correlation between the two experiments. The shape of the symbol shows how the sense-antisense counterparts overlap. [file gb-2010-11-3-r24-S8.pdf]

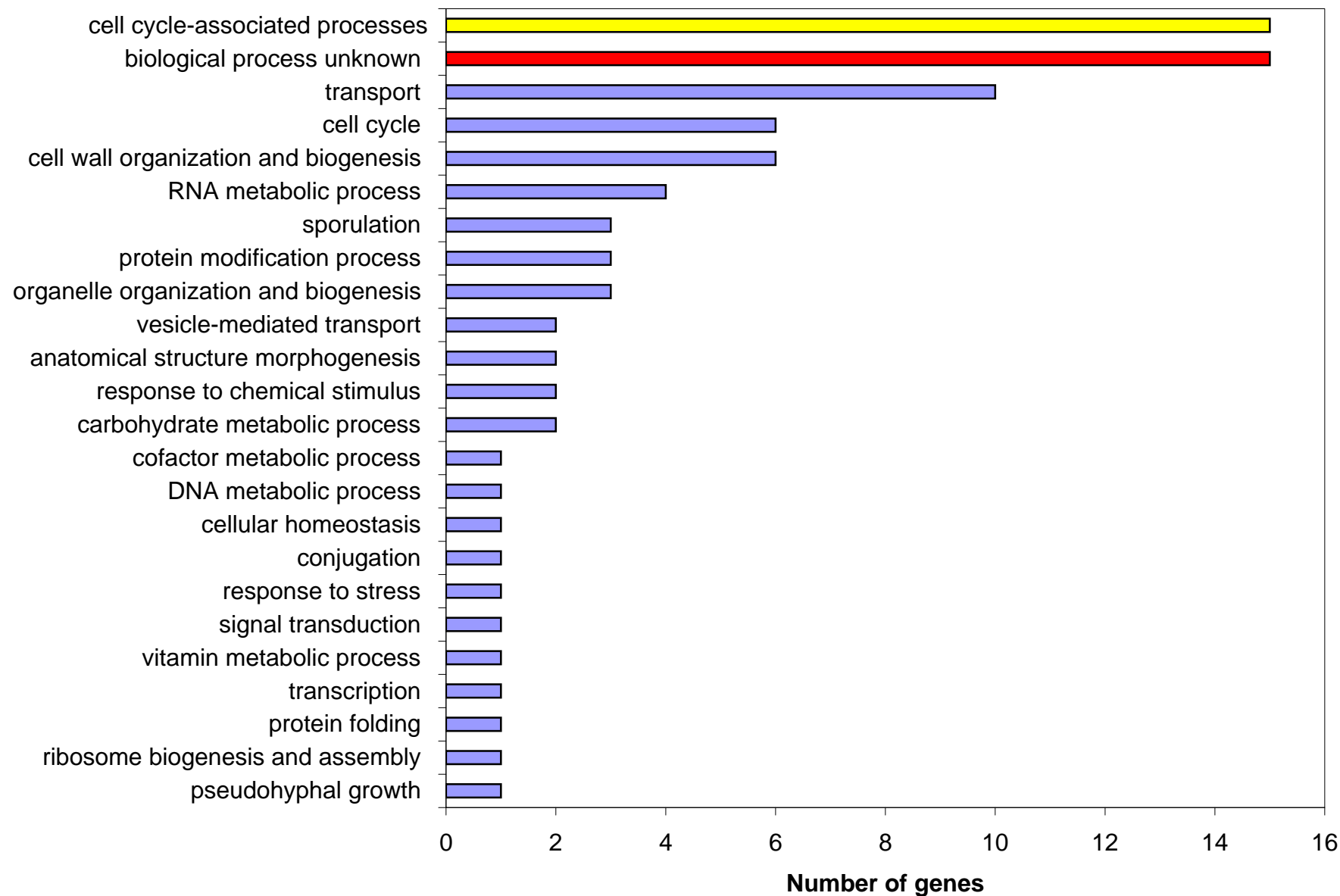

Supplement: Additional file 10 — A figure showing GO categories of the ORFs opposite cell-cycle-regulated antisense transcripts. The x-axis displays the number of genes and the y-axis shows the names of GO categories. [file gb-2010-11-3-r24-S10.pdf]

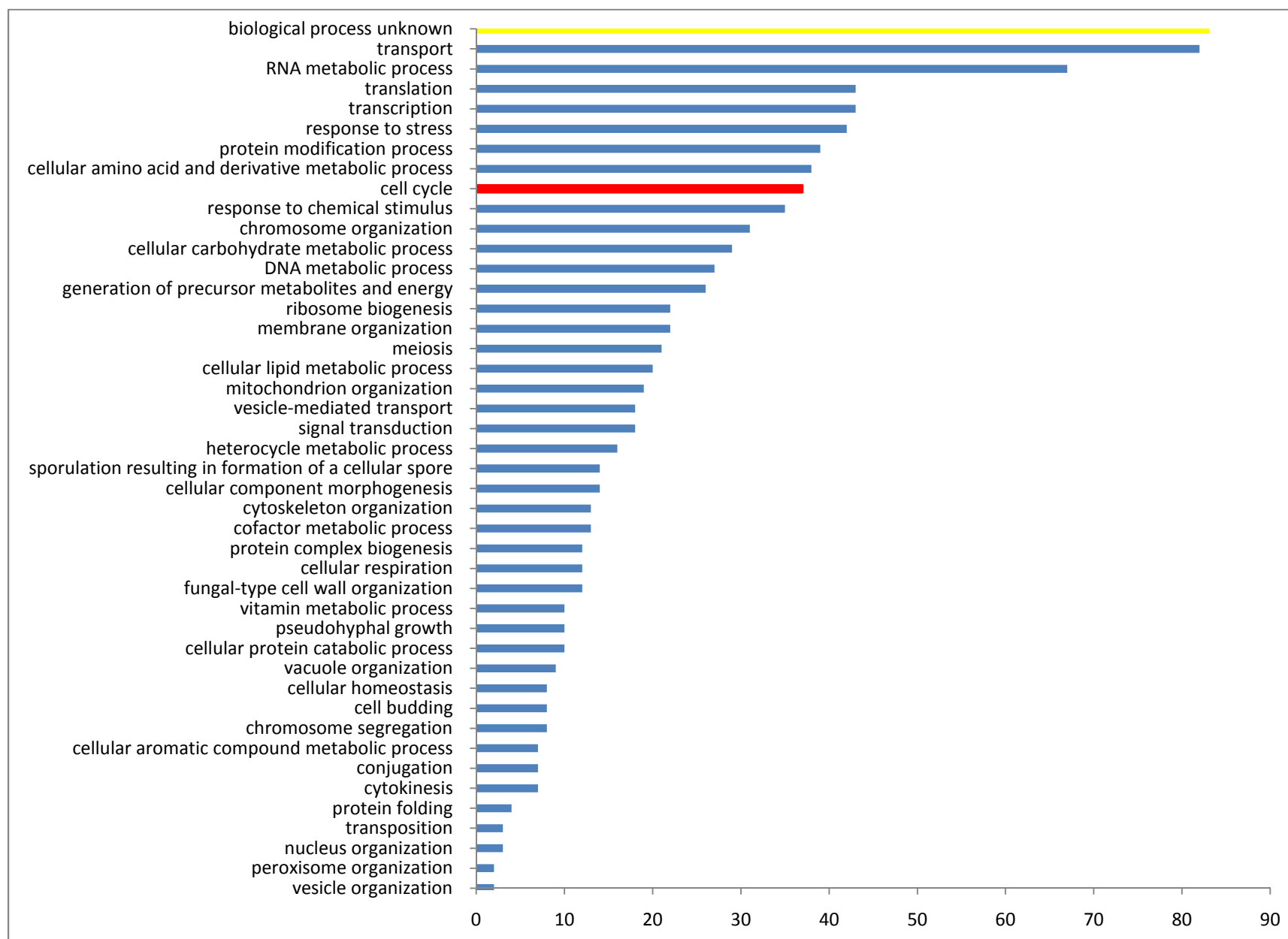

Supplement: Additional file 11 — A figure showing GO categories of 443 non-periodic ORFs opposite non-periodic antisense transcripts. The x-axis displays the number of genes and the y-axis shows the names of GO categories. [file gb-2010-11-3-r24-S11.pdf]

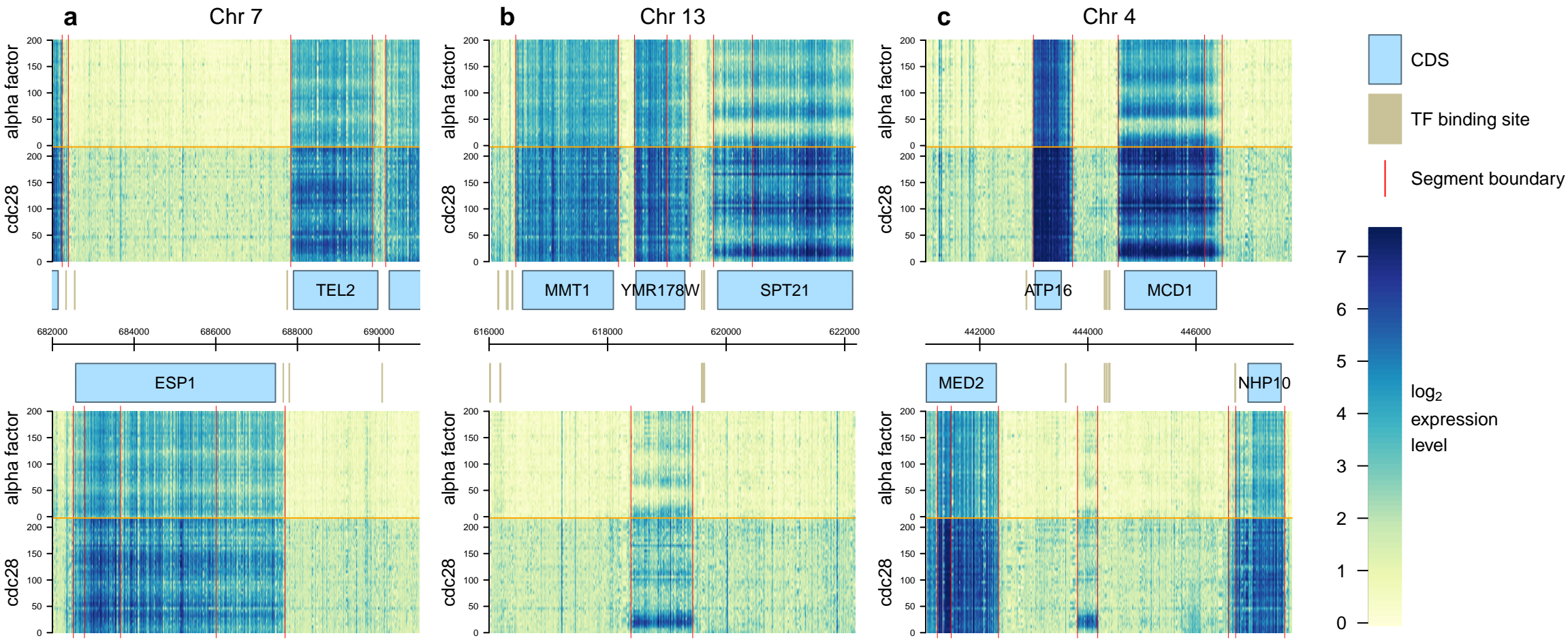

Supplement: Additional file 13 — A figure showing heatmaps of bi-directional expression of neighboring cell cycle-regulated genes that share transcription regulatory elements. (a) Two neighboring ORFs: TEL2 and ESP1. (b) ORF and an antisense transcript of the upstream protein-coding gene: SPT21 and antisense counterpart of YMR178W. (c) ORF and cycling unannotated intergenic transcript: MCD1and upstream cycling novel transcript. The heatmap plot is explained in the caption of Figure 3. [file gb-2010-11-3-r24-S13.pdf]

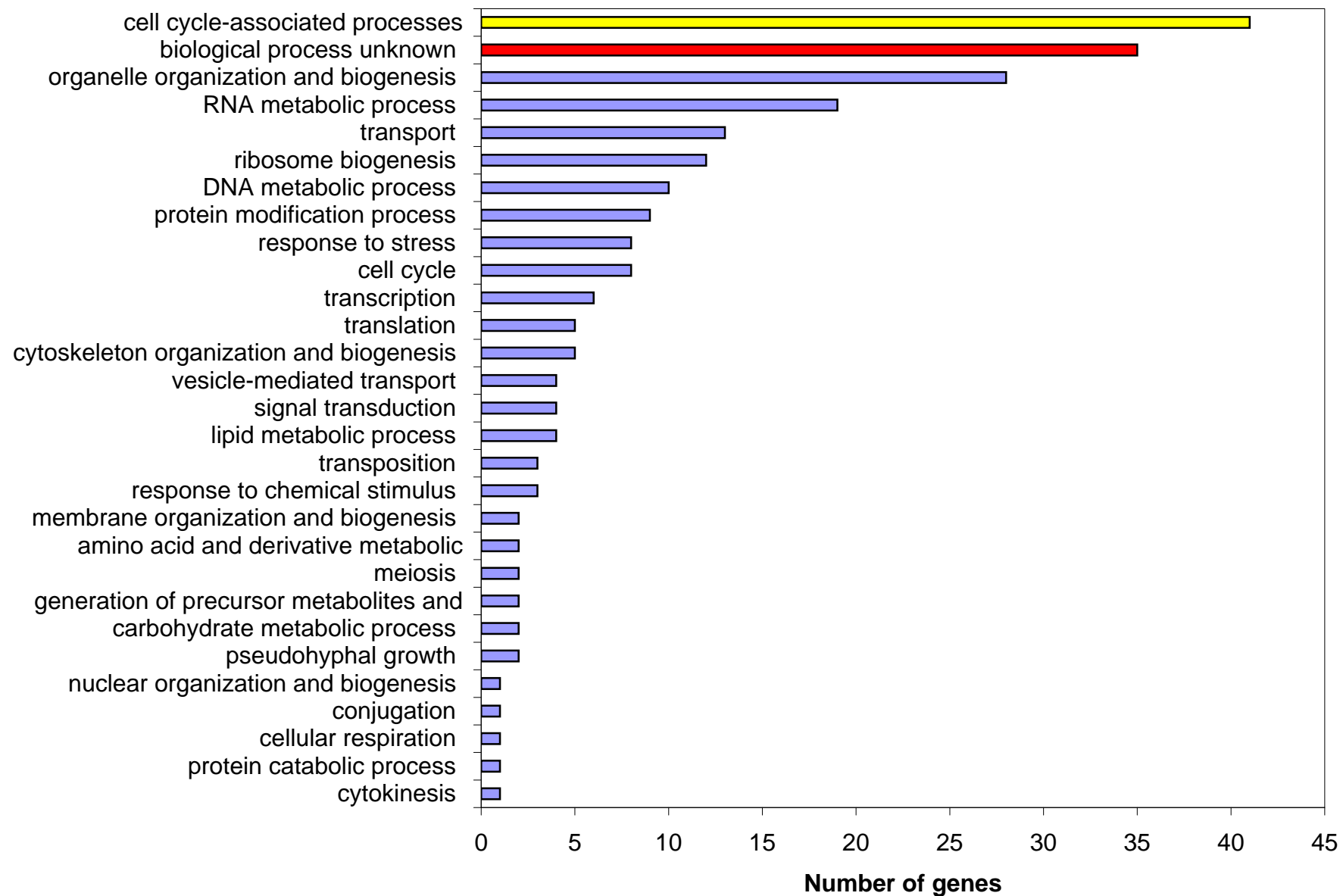

Supplement: Additional file 15 — A figure showing GO categories of 109 periodic ORFs unique to our dataset. The x-axis displays the number of genes and the y-axis shows the names of GO categories. [file gb-2010-11-3-r24-S15.pdf]

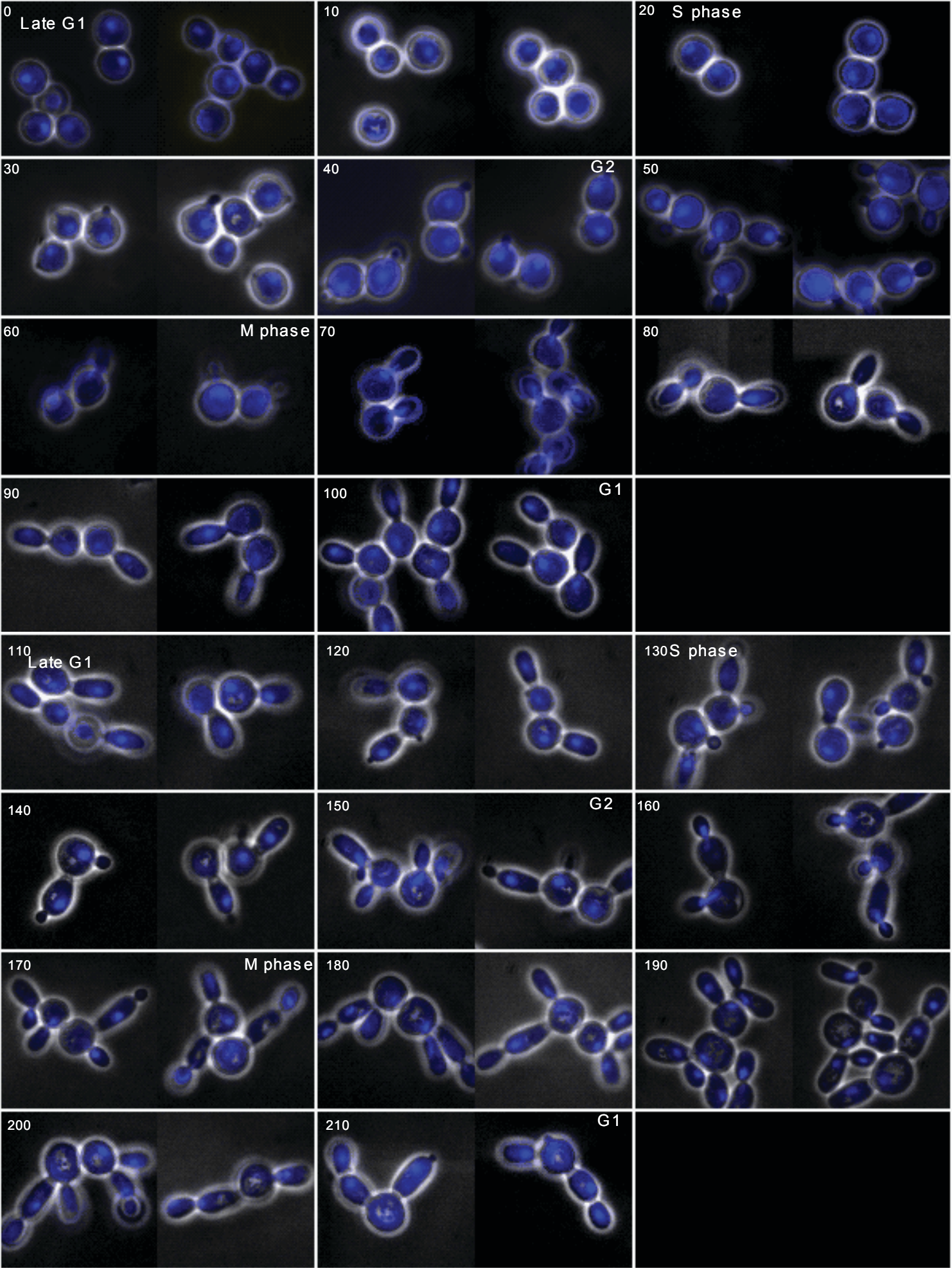

Supplement: Additional file 16 — A figure showing Hoechst nuclear staining of dividing cdc28-ts mutant cells. Control data displaying synchronous division of the yeast cells along with the cell cycle progression. Each image represents a gallery of approximately 10 to 20 representative cells that were chosen, for the respective time-point, from different fields of view. Criteria of choice were sharpness of the image and visibility of the bud; besides these, we aimed for random selection. [file gb-2010-11-3-r24-S16.pdf]
