# Supplementary material for: High-resolution transcription atlas of the mitotic cell cycle in budding yeast
Source: Genome Biol. 2010 Mar 1;11(3):R24. doi: 10.1186/gb-2010-11-3-r24 (PMC2864564; doi:10.1186/gb-2010-11-3-r24)
Supplement: Additional file 6 — A Word document providing supplemental data. The file provides additional information on the following sections: 1, Determination of the boundaries of the cell cycle phases; 2, Conservation analysis of non-coding RNAs; 3, Analysis of upstream regulatory elements for periodic unannotated transcripts; 4, UTR lengths; 5, Divergently transcribed periodic transcripts. [file gb-2010-11-3-r24-S6.doc]

**Supplemental Data**

Determination of the boundaries of the cell cycle phases.

We defined the phases in terms of percent of the cell cycle as follows:

G1 [5;35]

S [35;55]

G2 [55;70]

G2/M [70;80]

M [80;95]

M/G1 [95;5]

These numbers are in minutes, and were based on a careful analysis on when particular marker genes peaked in expression in the microarray data. For example, we know that the
genes of replication machinery as well as certain cyclins peak in expression in late G1 phase whereas the histones peak in expression during S phase; this allowed us to estimate the point when the cells transition between G1 phase and S phase. Similarly, we know M-phase cyclins as well as other proteins that peak in expression during M phase, which allowed us to estimate when M phase begins and ends. However, it is important to note that converting the fine-grained temporal data to a set of discrete phases is a simplification that we only used to help to discuss and understand the data.

Searching for functional conservation of non-coding RNAs.

To find clues to physiological functions of the newly discovered periodic ncRNAs, we overlapped them with the previously published regions of conserved RNA secondary structure [39]. Overlap of annotated RNA features to these regions was used as a control. Overall, 26 out of 27 rRNAs, 207 out of 299 tRNAs, and 34 of 77 snoRNA overlap such regions. In contrast, only 3 out of our 37 periodic antisense features overlap these genomic sequences, namely the ones opposite *YPR194C*, *YPR195C*, and *YMR181C*. For comparison, the percentage of coding transcripts overlapping regions of RNA conserved secondary structure is relatively small as well. Only 27 of the 252 non-cycling antisense (10.7%), 7.5% of cycling ORF transcripts, and none of the 11 cycling unannotated intergenic transcripts overlap such regions. The negative result of this analysis does, however, not exclude function: e.g., regulation by transcriptional interference or epigenetic modifications does not require conservation of RNA secondary structure.

Analysis of Upstream Regulatory Elements for Periodic Unannotated Transcripts

Phase-specific transcription factors mediate distinct waves of gene expression for protein-coding genes throughout the cell cycle.

To determine if the unannotated periodic transcripts are regulated by the same transcription factors (TFs) that drive cell-cycle progression of protein-coding genes [45, 47], we investigated their putative regulatory regions for known TF-binding motifs. For each of the 37 periodic antisense transcripts and 11 periodic intergenic transcripts (Table S3 and S1 in Additional files 7 and 3, respectively), we analyzed the transcribed region extended by 600 bp up- and down-stream. The promoter regions of 10 out of 48 periodic non-coding transcripts harbor putative TF-binding sites for Mbp1, which regulates the G1/S cell-cycle transition. Together with its cofactor Swi6, Mbp1 comprises the MBF (MCB-Binding Factor), which activates expression of the G1 cyclins, as well as genes involved in DNA synthesis and DNA repair, such as *CDC21*, *CDC8*, and *CDC9*. Of the 31 periodic non-coding transcripts that peak in late G1 or S phase, 10 had Mbp1-motifs, whereas only 2 of the remaining 17 contain this motif. These results suggest that the periodic expression of these transcripts is regulated by the same TFs as for known cycling genes.

UTR lengths.

The use of tiling arrays enabled determining the length of the 3’and 5’untranslated regions of ORFs. We confirmed our previous observation that 3’UTRs are longer than 5’UTR [11], also for the subset of cell cycle-regulated ORFs. However, we did not observe any difference between the UTR lengths of periodically expressed genes versus other genes.

Divergently transcribed periodic transcripts.

Progression through each phase of the cell cycle requires temporal coordination of multiple cellular processes. Hence the effectors functionally linked to different processes are co-expressed and in some cases regulated by the same transcription factors, which bind the regulatory sequence of a shared bidirectional promoter. In budding yeast, about 25 pairs of periodic genes, separated by fewer than 1500 bp, are divergently transcribed from opposite strands [28]. We investigated the transcription architecture, i.e. the orientation of 'nearby' transcripts to each other, for the periodic transcripts and compared it to that for all transcripts in the data. Two transcripts were considered a pair if the non-transcribed inter-transcript region between them was less than 400 bp long (this is an arbitrary cutoff).

Examples of periodic expression from putative bidirectional promoters are observed in our cell-cycle dataset for all transcript categories (Fig. S6 in Additional file 12). Six antisense and seven unannotated intergenic transcripts are co-expressed with an upstream ORF, and 29 pairs of adjacent ORFs are divergently transcribed (2 divergent ORF pairs escaped automatic detection due to alternative transcription start sites). More than 25% of the divergently positioned ORFs fulfill important functions in the cell cycle. Examples of several pairs of divergent genes that are involved in different processes important for cell cycle progression include *POL3–QRI1*, *ESP1–TEL2*, and *DPB2–YPR174C*, which are expressed in S phase, and *NBA1–NUF2*, expressed in G2 phase (see Table S5 in Additional file 16).

To test if the ncRNAs might regulate other genes in *trans*, we
performed a nucleotide BLAST search of all antisense and novel
unannotated intergenic transcripts against the rest of the genome.
However, we found no hits with an E-value below 1. This suggests that non-coding transcripts may exert an effect on transcription in the immediate surrounding or may be regulated by that themselves.
